# North Macedonia interprofessional dementia care (NOMAD) – personalized care plans for people with dementia and caregiver psychoeducation delivered at home by interprofessional teams

**DOI:** 10.3389/frdem.2024.1391471

**Published:** 2024-04-10

**Authors:** Gabriela Novotni, Marija Taneska, Antoni Novotni, Julia Fischer, Svetlana Iloski, Andrea Ivanovska, Vesna Dimitrova, Ljubisha Novotni, Miloš Milutinović, Boban Joksimoski, Ivan Chorbev, Shpresa Hasani, Vildan Dogan, Timo Grimmer, Alexander Kurz

**Affiliations:** ^1^Department of Cognitive Neurology and Neurodegenerative Diseases, University Clinic of Neurology, Skopje, North Macedonia; ^2^Faculty of Medicine, Ss. Cyril and Methodius University, Skopje, North Macedonia; ^3^Institute for Alzheimer's Disease and Neuroscience, Skopje, North Macedonia; ^4^University Clinic of Psychiatry, Skopje, North Macedonia; ^5^Department for Psychiatry and Psychotherapy, Center for Cognitive Disorders, Technical University of Munich, School of Medicine and Health, Klinikum rechts der Isar, Munich, Germany; ^6^Faculty of Computer Science and Engineering, Ss. Cyril and Methodius University, Skopje, North Macedonia

**Keywords:** dementia, post-diagnostic care, interprofessional memory teams, family caregivers, case management, nonpharmacological interventions

## Abstract

**Introduction:**

The increasing number of people living with dementia and its burden on families and systems particularly in low- and middle-income countries require comprehensive and efficient post-diagnostic management. This study aimed to explore the acceptability and efficacy of a multi-professional case management and psychoeducation model (North Macedonia Interprofessional Dementia Care, or NOMAD) delivered by mobile teams for people with dementia and their caregivers in North Macedonia.

**Method:**

We conducted a two-arm randomized controlled trial comparing the intervention with treatment as usual. Participants were recruited from 12 general practitioner (GP) offices in the Skopje region. The NOMAD intervention included the delivery of a personalized care plan over four home visits to dyads of people with dementia and their caregivers by a team including a dementia nurse and a social worker, in collaboration with GPs and dementia experts, and the introduction of a caregiver manual. We assessed caregivers' depressive symptoms, burden, and quality of life and the neuropsychiatric symptoms, daily living activities, and service utilization of people with dementia at baseline and follow-up; we also assessed the acceptability of the intervention by analyzing case notes and attendance rates.

**Results:**

One hundred and twenty dyads were recruited and randomized to either the control (*n* = 60) or the intervention group (*n* = 60). At follow-up, caregivers in the intervention group had, on average, scores that were 2.69 lower for depressive symptoms (95% CI [−4.75, −0.62], *p* = 0.012), and people with dementia had, on average, 11.32 fewer neuropsychiatric symptoms (95% CI [−19.74, −2.90], *p* = 0.009) and used, on average, 1.81 fewer healthcare services (95% CI [−2.61, −1.00], *p* < 0.001) compared to the control group. The completion of the home visits was 100%, but the intervention's acceptability was underpinned by relationship building, GP competencies, and resources to support families with dementia. There were no differences in the caregivers' quality of life and burden levels or daily living activities in people with dementia. NOMAD is the first case management, non-pharmacological, and multi-professional intervention tested in North Macedonia.

**Discussion:**

The trial showed that it is effective in reducing caregivers' depressive symptoms and neuropsychiatric symptoms in people with dementia and the burden on health and social care services, and it is acceptable for families. Implementing NOMAD in practice will require building primary care capacity and recognizing dementia as a national priority.

## 1 Introduction

Dementia is a significant public health and societal challenge that impacts individuals, families, societies, and healthcare and social systems. With the constant increase of the aging population, longevity worldwide, and, consequently, the number of people living with dementia (people with dementia), from over 50 million people with dementia in 2020 to 152 million in 2050 (Alzheimer's Disease International et al., 2020), comprehensive dementia management by both health and social care systems is of immense importance. This is underpinned by the recognition of dementia as a public health priority by the WHO and the WHO's call for nation-states to develop and implement national dementia policies (Neurology, 2023). However, out of 194 WHO members, only 39 had a national dementia plan adopted in 2023, most of which are high-income countries (HICs) (Alzheimer's Disease International et al., 2020). Low- and middle-income countries (LMICs) are unequally affected by dementia as, currently, 60% of people with dementia live in LIMCs, and by 2050, this percentage will rise to 71% (Alzheimer's Disease International et al., 2020). Additionally, more than 90% of people with dementia in LMICs are cared for at home, compared to 70% in HICs (Alzheimer's Disease International Karolinska Institutet, 2017). Despite this, the availability of services for people with dementia and specialist services is much lower in LMICs, with a gap of over 75% compared to HICs (Bruckner et al., 2010). Inequalities among countries are seen in each facet of the dementia management process, from awareness and early diagnosis and engaging biomarkers to treatment application facilities and infrastructure and creating collaborative post-diagnostic care models (Alzheimer's Disease International, 2022). These issues are exacerbated by the massive migration of young and middle-aged people and the “brain drain” observed in LMICs, which will eventually shift care provision for people with dementia to institutions and social services that are not adequately equipped for it (Mehrabian et al., 2019; Alzheimer Europe, 2023).

Not only people with dementia but also their family members, as informal and unpaid caregivers, are harmed by dementia mismanagement. Informal caregivers of people with dementia need to be supported by professionals as they otherwise experience high levels of stress, burden, and mental and physical health problems, as well as reduced ability to carry on their personal and professional duties (Galvin et al., 2014; Alzheimer's Disease Intrernational, 2015). More than a third of family caregivers experience depressive symptoms (Sallim et al., 2015; Cheng, 2017), a third experience feelings of pre-death or complicated grief (Crawley et al., 2022), and up to two-thirds experience symptoms of anxiety (Sallim et al., 2015; Cheng, 2017). Compared to people who care for family members with other conditions, dementia caregivers are significantly more stressed and suffer more serious depressive symptoms and physical problems (Pinquart and Sörensen, 2003a,b; Cheng, 2017). Caregivers' burden is affected by the symptoms of the person living with dementia, their level of dependence, the caregiver's gender and health, and their relationship with the person with dementia (Brodaty et al., 2014; Lindeza et al., 2020). This is exacerbated in LMICs compared to HICs due to the even greater lack of formal services and benefits for people with dementia and caregivers (Gauthier et al., 2022), as well as cultural and religious norms around caregiving (Thrush and Hyder, 2014).

### 1.1 Dementia in North Macedonia

North Macedonia is an LMIC (World Bank, 2023) with an estimated global prevalence of dementia of 28,279 in 2019, with the majority of people with dementia being cared for by family members in the community. The prevalence is expected to increase by 166% to 75,147 in 2050 (Nichols et al., 2022).

The current state of dementia care in North Macedonia is characterized by significant deficits regarding public awareness, diagnosis, treatment options, service provision, and research. Dementia is not recognized as in the national policy and research priorities, and there is a lack of dementia-inclusive initiatives and communities, protection of the legal rights of people with dementia, and availability of care services (Alzheimer Europe, 2023). Additionally, no specialized dementia referral centers or memory clinics offering interprofessional approaches exist (Alzheimer Europe, 2023).

During the past 4 years, the diagnostic protocol for dementia was improved by introducing Cerebrospinal fluid (CSF) biomarkers, but this was not accompanied by an improvement in the post-diagnostic management of dementia (Novotni, 2019). Currently, the treatment of dementia is exclusively pharmacological, with donepezil being the only fully funded treatment for dementia. There is no post-diagnostic support for people with dementia and their family caregivers in the form of education, respite, or support groups (Alzheimer Europe, 2023).

**Figure 1:**
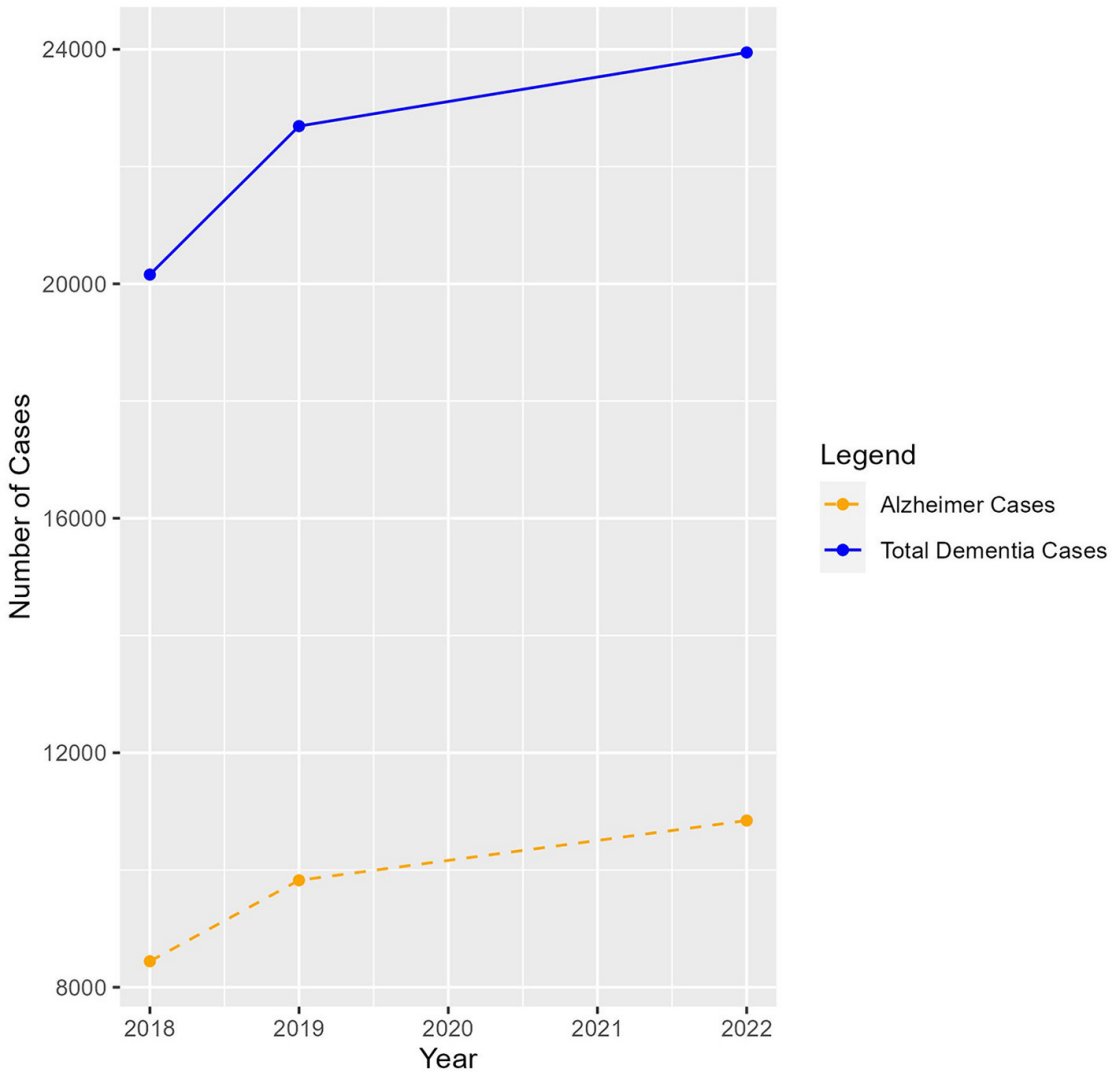
Dementia and Alzheimer's disease prevalence in Macedonia, 2018–2022.

According to the analyzed data from the electronic National Health System, the prevalence of people with dementia in Macedonia steadily increased from 20,158 in 2018 to 22,690 in 2019 to 23,947 in 2022 (Figure 1) (Velínov et al., 2024). Of the people with dementia, 8,444 (42.02%), 9,825 (43.31%), and 10,844 (45.25%) were diagnosed with Alzheimer's disease (AD) in 2018, 2019, and 2022, respectively. These data should further be analyzed as they may reflect not only a lack of adherence to treatment guidelines but also misuse of the AD International Classification of Diseases (ICD)-10 code to gain the minimal financial support granted by the Ministry of Labour Social Policy (2024). It indicated that the recommended treatment for AD—donepezil (prescribed to only 65% of the patients)—is under-prescribed and that antipsychotics (up to 26%) and benzodiazepines (up to 17%) are overprescribed (see the Appendix for an illustration of the trends in pharmacological treatment). The trend of prescribing antipsychotics has decreased globally to under 15%, with a marked increase during COVID-19 (Schnier et al., 2023), which was still lower than the numbers evidenced in North Macedonia. Due to the known risks of prescribing antipsychotics in people with dementia, non-pharmacological interventions should be offered as a safer alternative (Backhouse et al., 2016). This, unfortunately, as of now, is not an opportunity for people with dementia in North Macedonia due to the lack of post-diagnostic care. These numbers and insights emphasize the need for standardized dementia management guidelines, including post-diagnostic care and non-pharmacological treatments. Complying with dementia treatment guidelines and educating healthcare providers and informal caregivers should be recognized as top priorities in North Macedonia and should be included in future national dementia planning. A lack of awareness and education among healthcare professionals in understanding and proper diagnosis of dementia, supplemented by the stigma, on one side, and the normalization of the condition due to the normal aging process, on the other side, leads to underdiagnosis, misdiagnosis, or delayed diagnosis and treatment (Bradford et al., 2009), the consequences of which are missed treatment options, unnecessary institutionalizations, polypharmacy, medical complications, and increased healthcare costs (Rasmussen and Langerman, 2019).

With the lack of available and affordable care support services, the burden of dementia care falls on families who do so without any education or respite (Alzheimer Europe, 2023). A lack of support has been identified as the most significant predictor of a lower quality of life for dementia caregivers in North Macedonia, followed by high levels of burden and depression (Taneska, 2020). An additional problem is the increased migration among young and middle-aged Macedonians, which leads to a weakening of the capacity for both professional and family care and an increasing number of frail and older people living alone (International Organization for Migration, 2024).

### 1.2 The North Macedonia interprofessional dementia care project as a model of multicomponent, interprofessional dementia care

The project presented in this article was developed in view of the inadequate situation of dementia care provision in North Macedonia, with the aim of suggesting how to improve the situation by implementing and evaluating a multicomponent and interprofessional dementia post-diagnostic care model. Evidence indicates that dementia post-diagnostic care needs to encompass a range of interventions and support services aimed at addressing the multifaceted needs of individuals after a dementia diagnosis by an interprofessional team following a personalized care plan (Dreier-Wolfgramm et al., 2017; Heintz et al., 2020). This should involve the coordination of medical, social, educational, and psychological support to optimize the wellbeing of people with dementia and their families. Post-diagnostic dementia care plans should be person-centered, affordable, accessible, stigma-free, and culturally appropriate and should involve highly coordinated multi-professional activities to inform, educate, support, and guide people with dementia during the care receiver–caregiver journey by coordinating services across healthcare and community settings (Gauthier et al., 2022). Timely identification and management of the needs of people with dementia and their family caregivers regarding understanding dementia, psychological and emotional wellbeing, and practical support across the dementia journey have been identified as the key components of good post-diagnostic support (Bamford et al., 2021). This is accomplished by integrating support by care coordination, naming a point of contact, and managing transitions through regular and holistic reviews of the care plan and the needs of the caregiver to support physical health and planning for future needs.

Interprofessional collaborative models of dementia care provide several benefits, such as greater adherence to medical guidelines, more appropriate management of behavioral and psychological problems, augmented detection of comorbid conditions, fewer emergency and inpatient admissions, delayed patient institutionalization, fewer referrals to specialists, more use of community resources, reduced carer burden, and increased competence and confidence of professional care providers (Michalowsky et al., 2019; Heintz et al., 2020). They offer an integration of health and social care services and are cost-effective (Galvin et al., 2014; Wong et al., 2023).

In regions with barriers to access to services, mobile care teams are a promising way of delivering interprofessional collaborative care in the home. Models of primary care dementia support, care coordination, and home visits by multi-professional teams are consistently showing benefits for people with complex needs, including those with diabetes, heart disease, and dementia, as these models provide a collaborative approach to care and bring together general practitioners (GPs), nurses, social workers, and occupational therapists to deliver coordinated and holistic support to individuals – in our case people with dementia – and their informal caregivers in their home (Dreier-Wolfgramm et al., 2017; Michalowsky et al., 2019; Bamford et al., 2021). They can be upscaled by digitalization and the utilization of online platforms and remote communication (Dreier-Wolfgramm et al., 2017). Community-based dementia care models may help destigmatize dementia, provide social inclusion, and transform the community into a dementia-friendly environment.

Psychosocial interventions for people with dementia can be clinically and cost-efficient in the short and long term (Livingston et al., 2013, 2014, 2019; Cheng and Zhang, 2020). They reduce levels of anxiety and depression, improve the quality of life of caregivers and people with dementia, improve behavioral symptoms, and delay institutionalization (Dickinson et al., 2016).

The overall aim of the North Macedonia Interprofessional Dementia Care (NOMAD) study was to set up and evaluate an innovative post-diagnostic non-pharmacological dementia care model delivered at home by mobile interdisciplinary memory teams (MT) that complemented conventional care provided by GPs for people with dementia and their carers in North Macedonia. It was based on the insights from dementia care models in Germany from the Dementia Life- and Person-Centered Help in Mecklenburg-Western Pomerania (DelpHi-MV) (Thyrian et al., 2016) and the DemStepCare project (Bablok et al., 2021). In the DelpHi-MV project, specially qualified nurses (dementia care managers) conduct home visits and coordinate care in close collaboration with GPs, psychiatrists, neurologists, and nurses, as well as community care support points (Brodaty et al., 2014). To overcome the shortcomings in primary dementia care, such as delayed diagnoses, using therapies off guidelines, and frequent hospital admissions of people with dementia, the demStepCare project again (Bablok et al., 2021) offers a GP-based dementia care model. GPs are supported by case managers (specially trained nurses) and mobile teams consisting of a psychiatrist, a psychologist, and a social worker. While the main task of the case manager is to coordinate services, the mobile team primarily becomes involved in emergencies, for example, due to behavioral problems (Thyrian et al., 2016).

The aim of this study is to test the efficacy and acceptability of NOMAD: an intervention for family caregivers and people with dementia including psychoeducation and the creation of a personalized care plan delivered by mobile interprofessional teams in North Macedonia.

## 2 Methods

### 2.1 Study design

NOMAD is a two-arm cluster randomized controlled trial, evaluating the effectiveness of psychoeducation and individual, person-centered, and non-pharmacological treatment plans delivered by mobile interdisciplinary teams on the quality of life, burden, and depression in dementia family caregivers and resources utilization, activities of daily living (ADLs), and neuropsychiatric symptoms of people with dementia.

### 2.2 Recruitment

In the Skopje region, 20 GP offices were invited to take part in the study, through emails, letters, or phone calls, and 12 (60%) accepted. They were based in Skopje and its surrounding area, including rural areas (8 GP offices from the central region, 2 from rural areas, and 2 that cover both urban and rural areas surrounding Skopje).

#### 2.2.1 Problems with recruitment

There was a lack of interest from the GPs in participating in the study, mostly due to time constraints, high patient loads, and limited availability to be involved in extra activities and work with people with dementia. GPs spent considerable time selecting patients with dementia, many of whom lacked proper diagnosis according to the criteria and were diagnosed with dementia without following the diagnostic protocol (excluding reversible causes for dementia, neuropsychological evaluation, and neuroimaging). GPs, alongside dementia experts, had to conduct comprehensive examinations of patients' medical histories to ensure an accurate confirmation of dementia, which affected the timeline of the research.

### 2.3 Randomization

**Figure 2:**
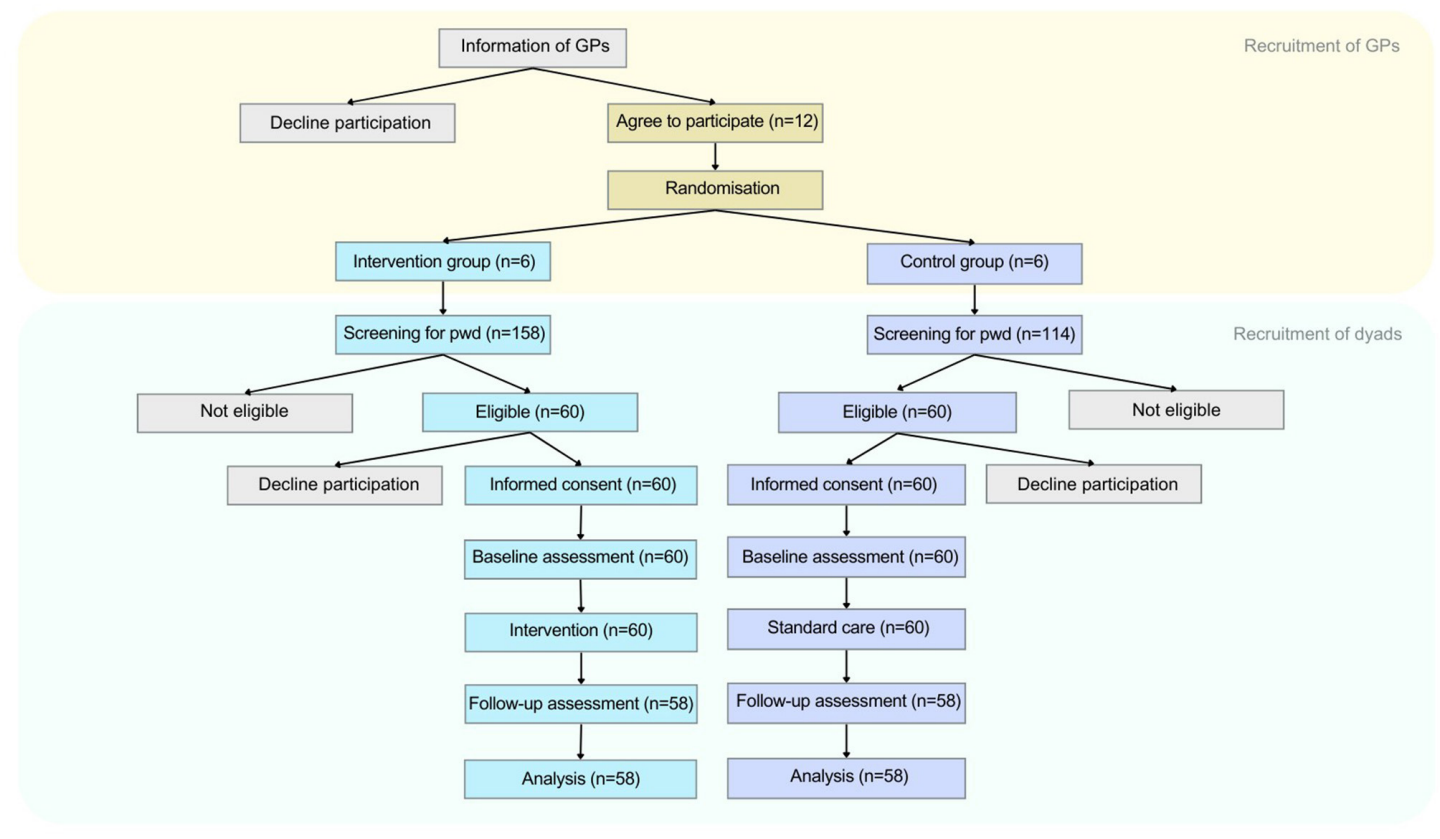
Participant flow.

We used cluster randomization to allocate GP offices to an intervention (*n* = 6) and a control group (*n* = 6) at a ratio of 1:1. The allocation was completed by an independent researcher using a computer-generated randomization system. Each GP was asked to enroll 10 people with dementia and their family caregiver within 3 months (i.e., ~3 per month). A screening-to-inclusion ratio of 2:1 was assumed, but it varied from 2:1 to 3:1 due to misdiagnosis (Figure 2). The researchers contacted participants after allocation to inform them about their allocation.

### 2.4 Participants

Eligible participants were dyads of people with dementia and a family caregiver registered with a GP in the Skopje region. We included only dyads in which the people with dementia were diagnosed with AD, vascular dementia, or frontotemporal dementia and who were supported by at least one family member for at least 5 h per week, regardless of whether they lived together. Participants with other or unspecified dementia diagnoses and caregivers who could not offer informed consent were excluded (including when they did not have guardianship to consent on behalf of the people with dementia when they were formally assessed as not having capacity). When the person with dementia was not formally assessed as not having capacity, we asked for consent directly.

### 2.5 Procedures

After confirming the accuracy of the diagnosis, GPs referred the eligible participants to the research team. An independent researcher contacted the participants over the phone to confirm eligibility, answer any outstanding questions, and obtain consent. When a guardianship [Ministry of Labour Social Policy. Family Act 5 (135)., 2024] was in place, the family caregiver consented on behalf of the person with dementia. The baseline assessment was carried out directly after obtaining consent or, if the participant preferred, at a later timepoint. All eligible participants consented to participate.

#### 2.5.1 Treatment as usual

Treatment as usual involved the standard pharmacological treatment mentioned earlier. After diagnosis, people with dementia in North Macedonia are offered pharmacological treatment (for AD, donepezil is fully compensated by the insurance fund while memantine is not), but there is no post-diagnostic care, psychoeducation, or support. The GP is the first point of contact for any consultations or changes in symptoms, and they refer the patients to secondary or tertiary care when necessary.

### 2.6 Assessment

All assessments were carried out with the family caregiver at baseline and after the last home visit over the phone. At baseline, we collected information about the family caregiver's and the person with dementia's sex and age, their relationship, their living situation, and the length of care.

Caregivers' depression was assessed with the PHQ-9 (Kroenke et al., 2001), a 9-item questionnaire which assesses the severity of depression based on DSM-IV criteria and its impact on daily functioning. The outcome measures were assessed at the two time points:

- The Bayer Activities of Daily Living (B-ADL) (Hindmarch et al., 1998) was used to assess the impairment of the activities of daily living of the person with dementia. The scale comprises 25 items, and it is completed by the caregiver.
- The quality of life of the family caregivers was assessed using the Caregiver Dementia-Related Quality of Life (C-DEMQOL) questionnaire (Brown et al., 2019), which comprises 30 items that measure 6 domains of quality of life.
- The level of burden in caregivers was assessed using the short form of the Zarit Burden Interview (ZBI) (Bédard et al., 2001), which consists of 12 items.
- The utilization of resources of the people with dementia and the family caregiver was assessed using an integrated measure: Resources Utilization in Dementia (RUD) instrument (Wimo et al., 2010), which consists of 70 items. The caregiver answered the questions that relate to their resource utilization in the last month and the resource utilization of the person with dementia.
- The neuropsychiatric symptoms of dementia were assessed using the Neuropsychiatric Inventory (NPI) (Cummings et al., 1994). It is a 12-item semistructured clinician interview answered by caregivers. Each of the 12 items corresponds to a specific domain in which the disturbance is rated.
- The acceptability of the intervention was determined by the number of visits completed with each dyad and analyzing the contents of the case notes of the MTs. An attendance of >50% indicated that the intervention was acceptable. We reviewed the case notes of the MTs to check for any barriers or facilitators to acceptability experienced on the field.

### 2.7 Blinding of participants

It was impossible to blind participants to their group allocation. They were asked not to disclose it or any details that would indicate their group allocation to the assessor. The two outcome assessors were blinded to the participants' group allocation.

### 2.8 Intervention

**Figure 3:**
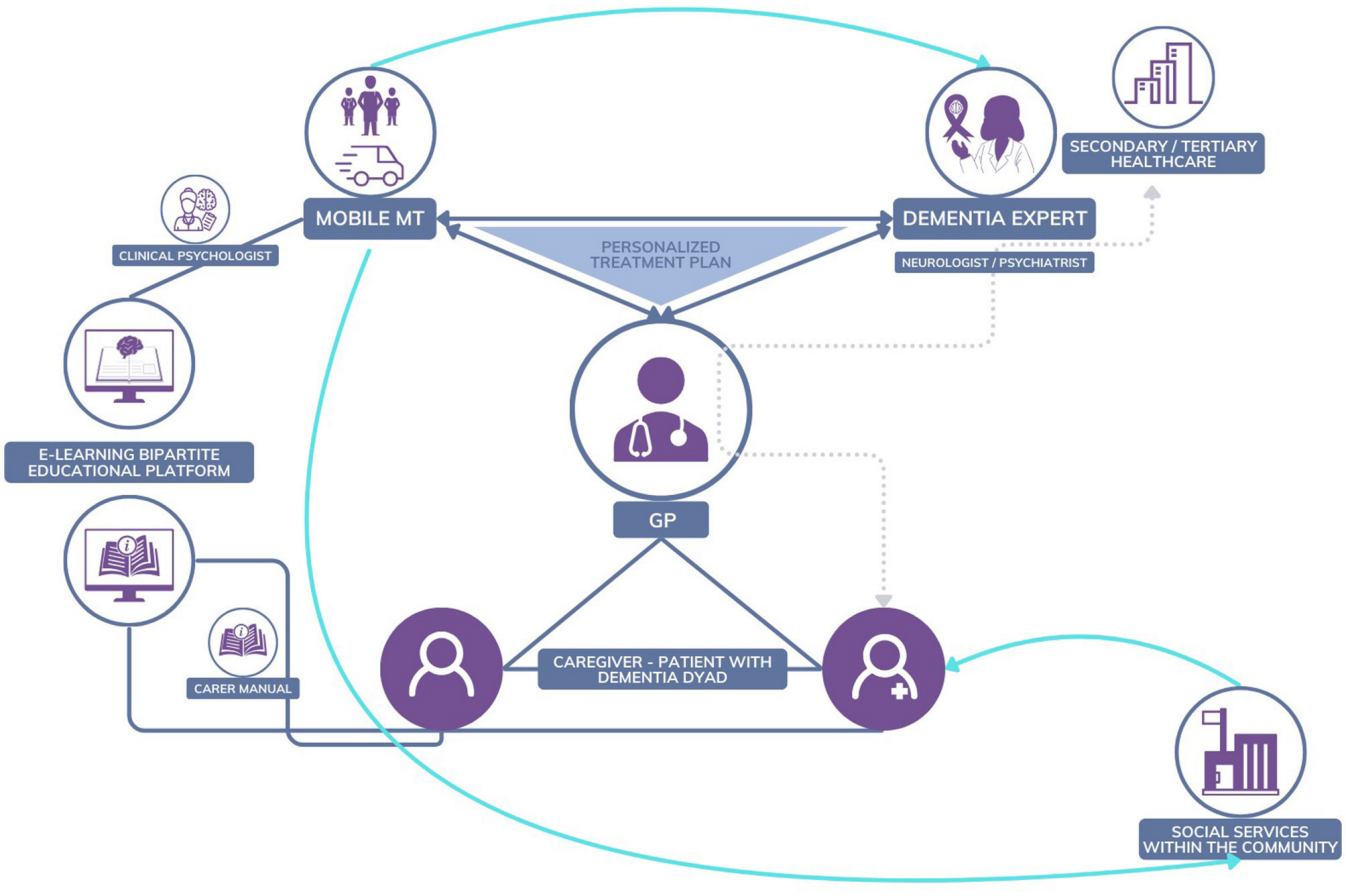
North Macedonia interprofessional dementia care model.

The NOMAD intervention combines methods of case management using a personalized care plan delivered by an interprofessional team and psychoeducation (Figure 3). The idea behind NOMAD was to develop and test the effectiveness of an innovative, interprofessional, collaborative, post-diagnostic, non-pharmacological dementia care model set up in North Macedonia. The model combines case management using a personalized care plan delivered by an interprofessional MT during four home visits and psychoeducation for dementia family caregivers. It is the first case management and non-pharmacological post-diagnostic intervention for people with dementia to be tested in North Macedonia. The MTs implement a systematic approach to address the diverse needs of individuals with dementia and their caregivers. This includes meticulously planning each visit and ensuring tailored support and interventions. Central to our approach is the integration of case management, and facilitating coordinated access to medical, social, and community resources. Additionally, psychoeducation is provided to family caregivers, equipping them with essential knowledge and skills to manage dementia-related challenges effectively. Delivering care and education through home visits, close monitoring, flexibility, and responsiveness are the key principles guiding the MTs' approach. This comprehensive model aimed to improve outcomes for people with dementia while enhancing family caregivers' wellbeing and support.

*Home visits* (Table 1). *Three* MTs, each assigned to two GP offices, completed four home visits over 6 months. The multi-professional memory teams combined a team of a dementia nurse and a social worker with previous experience in working with people with dementia and carrying out home visits with people with mental health problems. There were 6 MT members, 1 man and 5 women, with an average age of 40.17 and an average work experience of 18.17 years. The families could contact the MTs between sessions for any emerging questions. The MTs created and modified the treatment plan in collaboration with the dementia expert and the patient's GP during the whole intervention period.

**Table 1:** Description of home visits.

**Home visit**	**Description**
1	*Comprehensive assessment, development of personalized treatment plans, and coordination of treatment plans with general practitioner* During the first home visit, the memory team (MT) assessed the needs of the person with dementia based on the comprehensive assessment and introduced the caregiver manual and the educational platform. After the first visit, MTs created a personalized treatment care plan for each dementia dyad in collaboration with the general practitioner (GP) and the dementia expert. The plan was developed according to a comprehensive assessment covering 12 domains of needs (communication, mobility, toileting, and continence, showering and dressing, skin care and pressure, nutrition and hydration, sleep, medication, special care needs, risk, social needs, and behavior). Each domain had several subdomains and options to choose from and space for additional notes. The assessment tool was developed by the dementia experts and clinical psychologists. Based on the assessment, the MTs created a personalized care plan with specific actions, including referrals to health and social care services, addressing the identified needs in collaboration with the GP and the dementia expert.
2	*Implementation of the treatment plan and initiation of appropriate community support* The MT worked collaboratively with the person with dementia and the caregiver to implement and adapt the individualized treatment plan developed in the previous phase. This included referrals for initiating or reviewing pharmacological intervention when appropriate, implementing cognitive and behavioral strategies, and incorporating lifestyle modifications to address each person with dementia's unique needs and goals (e.g., environmental modifications, behavioral interventions, etc.) The MT also provided information on social support opportunities. After introducing the care plan, the MT closely monitored the progress of the person with dementia and the family caregiver and the adequacy of the care plan during subsequent visits and phone calls. Feedback from the person with dementia and the family caregiver was sought to identify any challenges or areas requiring adaptation. The MT remained flexible and responsive, making the necessary modifications to treatment plans and support services to optimize outcomes and respond to evolving needs.
3	*Assessment of treatment progress, coordination with the GP and dementia expert, and adjustment of treatment plan* The MT reassessed the needs of the people with dementia, evaluated the progress of the treatment plan, and identified any changes in the person with dementia's conditions. Feedback from the person with dementia and the caregiver was obtained to gain insights into their experiences and perspectives regarding the effectiveness of interventions. When necessary, adjustments were made in collaboration with the dementia expert based on the reassessment.
4	*Final assessment of treatment progress, coordination with the general practitioner, and adjustment of the treatment plan* The MT conducted the final review of the care plan and created actions to maintain it in the future. Feedback from the person with dementia and the family caregiver was obtained to gain insights into their experiences and perspectives regarding the impact of treatment. Based on the final assessment findings, the MT recommends adjustments to the treatment plan to optimize outcomes and its future sustainability and address needs and challenges. This involves refining non-pharmacological interventions or enhancing support services to better meet the evolving needs of the patient and the caregiver.

#### 2.8.1 Educational e-learning platform^1^

NOMAD offers an innovative bipartite e-learning platform used during the preparatory phases to educate GPs and MT members and offer educational material to family caregivers. It was uniquely developed for the NOMAD project and was inspired by the Innovation for Dementia in the Danube Region model;^2^ it serves a double function: to entail educational materials for the professionals involved in the project and provide an e-version of the caregiver manual for participants who prefer an online version. The platform has been meticulously crafted to be user-friendly for all professionals across the health and social fields to enrich their competence and expertise in dementia knowledge and care. Through interactive modules, training materials, and case studies, professionals and carers can expand their understanding of dementia care best practices and refine their skills. The NOMAD e-learning platform has educational content tailored to complement occupation-specific training based on modern didactic concepts but upgraded with a carer manual. To our knowledge, the e-learning platform designed for the NOMAD purposes is the first of this kind of educational tool in North Macedonia, providing access to knowledge to both medical professionals and family caregivers of people with dementia in the country.

The model proposes that case managers bridge the gap between primary and secondary care. They are based in the secondary care services, and they are allocated GP services covering a given geographic area.

The *manual for family caregivers* covered information on recognizing and understanding dementia; treatment opportunities, portrayed by culturally appropriate case studies; and managing behavioral and psychological symptoms, carer self-care, and local support availability. It was assembled by health and social care professionals working with people with dementia with previous experience in developing the content of the Innovation for Dementia in the Danube Region^3^ project, which was codesigned with local experts from the Balkan region. Our team personalized the content and case studies based on their experience, with the consideration that the content was culturally attuned. This was the first time that this intervention was piloted in practice. It comprises 50 pages in total and was offered as a printed paper version and in an online format (e-platform developed for the project). Each family who opted to access the educational materials through an e-platform was given a unique log-in. *The caregivers' manual aims to strengthen the self-help potential of family caregivers*.

#### 2.8.2 Training and supervision

At the beginning of the NOMAD project, MTs and GPs from the intervention group attended four online training sessions (lasting 90–120 min) carried out by local dementia experts, including

- Introduction to the procedures of the intervention and creating personalized care plans.
- Recognition and screening of dementia (GPs).
- Information on understanding dementia and treatment opportunities, including non-pharmacological treatment options, during an educational course in person and online using the e-learning platform.
- Person-centered approaches in dementia care.

Throughout the NOMAD project, the MT members had biweekly consultation sessions with the dementia expert.

### 2.9 Data analysis

#### 2.9.1 Participants demographics and outcomes

All data were imported into a database. Total scores for the C-DEMQOL were calculated using the C- DEQMOL Score Calculator (V 1.0) by the Center for Dementia Studies, Brighton and Sussex Medical School (Banarjee, 2019). The score for the total number of services used was derived as the sum of the total visits to healthcare services included in the Patient HealthCare Resource Utilization section of the RUD, which includes nights spent in a hospital; visits to the emergency healthcare center, GP, geriatrician, neurologist, psychiatrist, physiotherapist, occupational therapist, social worker, and/or psychologist; and services from the district nurse, a home help/healthcare assistant, Meals on Wheels, a daycare, transportation (care-related), and other healthcare professionals and services. The rest of the total scores were manually calculated following the instruction manuals. All the analyses were done in RStudio version 2023.06.1 + 524 using R version 4.2.2 (R Core Team., 2020). Descriptive statistics were obtained to get an overview of the data using the psych package (Revelle, 2023). The central tendency, dispersion, and distribution of age and scores on the B-ADL, the NPI, the Patient Health Questionnaire−9 (PHQ-9), the ZBI, the C-DEMQOL, and the RUD were analyzed for all participants, as well as each subset of the data, including pretest and posttest scores across treatment groups.

To test the efficacy of the intervention, we used random-effects multivariate regression models adjusted for age, gender, and baseline outcome score using the lme4 (Bates et al., 2015) and lmerTest (Kuznetsova et al., 2017) packages. All graphical representations were created using ggplot2 (Wickham, 2016). The packages dplyr (Wickham, 2020) and tidyverse (Wickham et al., 2019) were also used to manipulate the data.

#### 2.9.2 Acceptability of the intervention

We reviewed the field notes taken by the MTs to identify themes regarding the acceptability of and reactions to the intervention, focusing on the affective attitudes of the participants (Sekhon et al., 2017) using a deductive approach for thematic analysis (Braun et al., 2018). One researcher reviewed, coded, and merged the codes into barriers or facilitators to acceptability. Additionally, we interviewed the members of the MTs to understand their experiences of delivering the session; these results will be published elsewhere. We identified the most common actions taken by the MTs from the field notes, according to the needs identified in the care notes and the phone consultations between visits.

#### 2.9.3 Ethics

Before recruitment and data collection, the study was approved by the Ethical Committee of the Medical Faculty at the University Ss Cyril and Methodius in Skopje, North Macedonia (Ref Number:03-1260/5). A written informed consent procedure with the legal proxy of the people with dementia was the basis for study participation. The study was conducted in compliance with European data protection guidelines.

Following the completion of the trial and for the purpose of this article, we submitted an application for access to the Macedonian e-health data regarding patients diagnosed with dementia to the Department for E-Health at the Ministry of Health (www.mojtermin.mk) and were granted permission in January 2024.

## 3 Results

### 3.1 Delivery

#### 3.1.1 Intervention

The intervention was conducted from November 2022 to August 2023. The average duration of the home visits by the MTs was 1 hour, and the average time between each home visit was ~46.3 days (Table 2). The average time between the first and last home visit was 142.5 days (~ 4.7 months).

**Table 2:** Duration of home visits.

**Visits**	**First and second**	**Second and third**	**Third and fourth**
Average time between visits in days	64.9	41.0	36.5

The following actions were taken by the MTs to address the identified needs from the care plans:

- Lifestyle interventions and home safety information. MTs provided home safety suggestions for potential hazards and risks in the home environment. They offered recommendations for modifications or adjustments to improve safety and reduce the risk of accidents or injury to the individual with dementia (e.g., adding a bell to the door that notifies other family members if the person with dementia is leaving the house).
- Arranging medical appointments and appointments with social work services for financial compensation
- Arranging more support (e.g., involving more family members in the care when identified that the primary caregiver is struggling to cope)
- Self-care for caregivers
- Information and education. Caregivers and other family members often require information and education about dementia, including its symptoms, but mostly about progression and management strategies. They needed guidance on how to cope with challenging behaviors, communication difficulties, and changes in the person's abilities.

#### 3.1.2 Phone consultations

The investigation pertains to a series of phone calls received over a specified period, totaling 17 calls from 12 participants (20%) (2 participants had 2 calls and 1 had 3). Each call ranged from 10 to 30 min in duration, covering various subjects and inquiries. The MTs received phone calls mostly regarding consultations for new situations arising during dementia care.

A common reason for caregivers contacting the MTs was to facilitate appointments with healthcare providers, particularly doctors. Furthermore, caregivers frequently reached out to arrange appointments with regional departments of the Centers for Social Protection in their respective municipalities. These appointments were essential for accessing social support services and resources, such as financial assistance (Table 3).

**Table 3:** Breakdown of phone calls received by the memory teams.

**Reason for call**	**Percentage of total calls**
Scheduling medical examinations	47.1
Organizing quick appointments with a specialist	11.8
Inquiring about financial support due to dementia	29.4
Dealing with anxiety, insomnia, and stress	11.8

### 3.2 Acceptability

#### 3.2.1 Attendance

The attendance rate was 100%, with all 60 families completing all the sessions.

#### 3.2.2 Field notes analysis

##### 3.2.2.1 Acceptability for participants

The analysis of field notes indicated that generally, participants had a welcoming attitude toward the MTs, although some barriers were identified due to a lack of trust and unfamiliarity with the model. Participating families were unfamiliar with this concept of care, and some felt apprehensive about welcoming the MTs into their homes. With a few families, the MTs had to meet at the GP's office first before the initial home visit, and more restrained attitudes were particularly emphasized during the first home visit. In some families, the MTs had to additionally explain the purposes of their visits and reassure them that they were not aimed at monitoring or controlling how they care for their family members. After the first home visit, the MTs recorded a noted change in the attitude of the families, which they attributed to the rapport they built during the first visit and the recognition of the families that this would be helpful for them. After the first home visit, there were no occurrences of hesitance in welcoming the MTs, and the opposite was noted. Most participants (83.3%) opted for the printed version of the caregiver manuals.

One finding derived from our observations was the evident inclination of study participants toward traditional paper-format manuals. Despite the accessibility of digital resources via the online platform, participants consistently articulated a preference for printed materials.

##### 3.2.2.2 Acceptability for GPs

Contrary to the planned delivery of the intervention, the involvement of the GPs in creating and following up the care plans was much lower. Besides the training they attended at the beginning of the study and the access to the e-platform, a lack of confidence in supporting people with dementia, and restricted availability owing to patient overload kept them away from frequent involvement in the personalized treatment planning with the MT, and the dementia expert in part compensated for their role.

### 3.3 Participants

#### 3.3.1 Recruitment flow

Of the 272 participants who were invited to take part, 120 consented (Figure 2).

#### 3.3.2 Participants demographics

The demographic and baseline clinical characteristics are presented in Table 4. Two participants were lost to follow-up in each group, so the final sample consisted of 116 participants. Caregivers' average age was 58.9 years (*SD* = 13.5), the majority were women (56%), cared for a parent (47%), and lived with the person with dementia (78%). Most participants were Macedonian (79%) and did not have children living in the household (60%), and only 17% did not have other family caregivers involved in the care. The average age of people with dementia was 74.6 (*SD* = 7.8), and the most common diagnosis was AD (92%).

**Table 4:** Demographics of participants.

**Characteristic**	**Control group, *n* = 58**	**Intervention group, *n* = 58**	**Total sample, *N* = 116**
**Caregiver, mean (** ***SD*** **)**			
Age	56.8 (11.0)	61.1 (15.4)	58.9 (13.5)
**Person with dementia, mean (** ***SD*** **)**			
Age	74.4 (7.8)	74.8 (8.1)	74.6 (7.8)
**Gender**, ***N*** **(%)**			
**Caregiver**			
Male	24 (41.4)	27 (46.6)	51 (44.0)
Female	34 (58.6)	31 (53.4)	65 (56)
**Person with dementia**			
Male	22 (37.9)	20 (34.5)	48 (41.4)
Female	36 (62.1)	38 (65.5)	68 (58.6)
**Nationality**, ***N*** **(%)**			
Macedonian	43 (74.1)	49 (84.5)	92 (79.3)
Albanian	15 (25.9)	4 (6.9)	19 (16.4)
Other	–	5 (8.6)	5 (4.3)
**Place of living**, ***N*** **(%)**			
Urban	50 (86.2)	43 (74.1)	93 (80.2)
Rural	8 (13.8)	15 (25.9)	23 (19.8)
**Dementia diagnosis**, ***N*** **(%)**			
Alzheimer's disease	52 (89.7)	55 (94.8)	107 (92.2)
Vascular dementia	6 (11.3)	2 (3.5)	8 (6.9)
Frontotemporal dementia	–	1 (1.7)	1 (0.9)
**Relationship of the caregiver to the person with dementia**, ***N*** **(%)**			
Spouse	22 (37.9)	28 (48.3)	50 (43.1)
Child	33 (56.9)	22 (27.9)	55 (47.4)
Other	3 (5.2)	8 (13.8)	11 (9.5)
**Number of other caregivers involved**, ***N*** **(%)**			
0	3 (5.2)	17 (29.3)	20 (17.2)
1	14 (24.1)	18 (31.0)	32 (27.6)
2	16 (27.6)	15 (25.0)	31 (26.7)
3+	25 (43.1)	8 (14.7)	33 (26.5)
**Number of children in the household**, ***N*** **(%)**			
0	31 (53.5)	35 (60.3)	66 (56.9)
1	10 (17.2)	10 (17.2)	20 (17.2)
2	8 (13.8)	6 (10.3)	14 (12.0)
3+	9 (15.5)	7 (12.2)	16 (13.9)
**Living together**, ***N*** **(%)**			
Yes	38 (65.5)	52 (89.7)	90 (77.6)
No	20 (34.5)	6 (10.3)	26 (22.4)
**Clinical scales, baseline values, mean (** ***SD*** **)**			
Depressive symptoms measured by the PHQ-9	13.0 (9.4)	11.6 (5.5)	12.3 (6.3)
Caregivers' burden measured by the ZBI	17.6 (9,4)	19.5 (7.7)	18.5 (8.6)
Caregivers' quality of life measured by the C-DEMQOL	94.2 (19.1)	95.3 (18.0)	94.7 (18.5)
Neuropsychiatric symptoms of dementia measured by the NPI	41.0 (28.0)	27.8 (28.8)	34.4 (20.0)
Activities of daily living in dementia measured by ADLs	7.1 (2.7)	6.8 (2.6)	7.0 (2.6)

PHQ-9, Patient Health Questionnaire−9; ZBI, Zarit Burden Interview; C-DEMQOL, Caregiver Dementia-Related Quality of Life questionnaire; NPI, Neuropsychiatric Inventory; ADL, activity of daily living.

The age of the people with dementia was similar in both groups, but caregivers in the intervention group were ~4 years older than those in the control group. In both groups, there were more female caregivers and people with dementia. The intervention group had more spouses (48% vs. 38%) and more commonly lived with the person with dementia (90% vs. 66%) compared to the control group. By comparison, 43% of the participants in the control group had three or more other caregivers involved in the care, whereas in the intervention group, this was the case for only 8% of the participants. In the intervention group, nobody was diagnosed with Frontotemporal dementia (FTD) or from a non-Macedonian or Albanian nationality. The clinical characteristics were balanced between the groups, except for neuropsychiatric symptoms of dementia, which were slightly higher in the control group. According to the average B-ADL score (~7 for both groups), the majority of people with dementia were in the moderate to severe dementia stage.

#### 3.3.3 Outcomes for persons living with dementia and family caregivers

##### 3.3.3.1 Family caregiver outcomes

**Figure 4:**
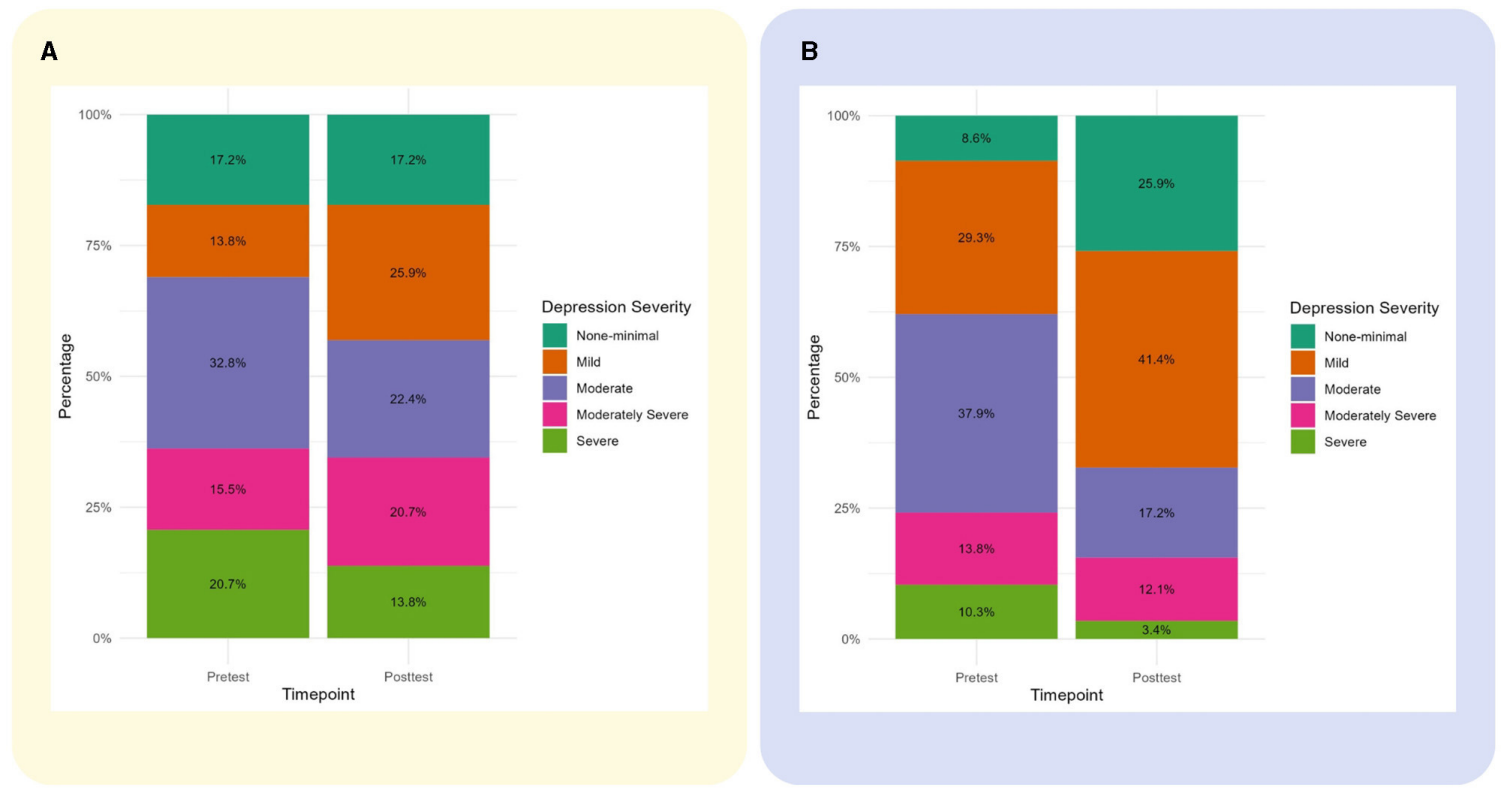
Changes in caregivers' depressive symptoms severity from baseline to follow-up. **(A)** Changes in the control group. **(B)** Changes in the intervention group.

We found a significant reduction in depression in family caregivers at T2. After adjusting for gender, age, and baseline depressive symptoms, the caregivers in the intervention group had an average of 2.69 (95% CI [−4.75, −0.62], *p* = 0.011) fewer depressive symptoms compared to the control group. There were no significant differences between the groups in the levels of caregiver burden (mean difference: −1.63, 95% CI [−4.85, 1.59], *p* = 0.317) and the quality of life (mean difference = 3.15, 95% CI [−3.47, 9.77], *p* = 0.34), but in the intervention group, the caregivers' burden was lower and the quality of life was higher (Table 5, Figure 4).

**Table 5:** Analysis of the effect of the intervention on the outcomes for caregivers and people with dementia.

**Outcome measure**	**Intervention group**	**Control group**	**Unadjusted effect size [95% CI]**	***p*-value**	**Adjusted effect size [95% CI]**	***p*-value**	***N***
**Caregiver outcomes**							
PHQ-9	8.31 (5.52)	11.38 (6.71)	−3.07 [−5.33, −0.81]	0.008	−2.69 [−4.75, −0.62]	0.011	116
ZBI	18.28 (10.18)	18.14 (9.91)	0.14 [−3.68, 3.95]	0.943	−1.63 [−4.85, 1.59]	0.317	116
C-DEMQOL	97.74 (22.56)	95.05 (19.69)	2.69 [−5.10, 10.48]	0.495	3.15 [−3.47, 9.77]	0.347	116
**People with dementia outcomes**							
NPI	22.43 (26.27)	40.64 (27.07)	−18.21 [−28.02, −8.40]	< 0.001	−11.32 [−19.74, −2.90]	0.009	116
RUD	2.09 (2.93)	0.24 (0.54)	−1.84 [−2.62, −1.07]	< 0.001	−1.81 [−2.61, −1.00]	< 0.001	116
ADL	7.40 (2.30)	7.92 (2.50)	−0.52 [−1.41, 0.36]	0.243	−0.36 [−0.89, 0.16]	0.175	115

PHQ-9, Patient Health Questionnaire−9; ZBI, Zarit Burden Interview; C-DEMQOL, Caregiver Dementia Related Quality of Life; NPI, Neuropsychiatric Inventory; RUD, Resources Utilization in Dementia; ADL, activity of daily living.

##### 3.3.3.2 People living with dementia outcomes

We found a significant reduction in neuropsychiatric symptoms and healthcare services used by people with dementia at T2. After adjusting for gender, age, and baseline neuropsychiatric symptoms, the people with dementia in the intervention group had an average of 11.32 (95% CI [−19.74, −0.90], *p* = 0.009) fewer neuropsychiatric symptoms and used, on average, 1.81 fewer healthcare services (95% CI [−2.61, −1.00], *p* < 0.001) compared to the control group. There was no significant difference between the groups in their ADLs (mean difference −0.36, 95% CI [−0.89, 0.16], *p* = 0.175) but in the intervention group, their impairment was lower (Table 5).

## 4 Discussion

This article provides an overview of the acceptability of the NOMAD intervention for families affected by dementia and its efficacy in improving healthcare outcomes and addressing factors contributing to the quality of life of family caregivers and people with dementia in North Macedonia. It combined case management by multi-professional teams over four home visits and caregivers' psychoeducation through a physical and an online version of a caregiver manual.

### 4.1 Feasibility and acceptability of the intervention

During the NOMAD study period, the e-learning platform was not used by the family caregivers as often as expected as they preferred the printed paper version of the carer manual, which is understandable as the mean age of the caregivers' group is 58.9, and younger generations are more inclined to accept and utilize e-learning. It also reflects the low usage of digital platforms among the general population in North Macedonia as only 1.5% of the population was registered to the government's e-services, and obtaining educational information is the least common use of the Internet (Increasing Civil Engagement in the Digital Agenda., 2021). Besides the widespread use of the Internet, it is still mainly used for communication and social media, and more refined digital skills are lacking (Increasing Civil Engagement in the Digital Agenda., 2021). This was reflected in Ho et al. (2015) study that showed that generally younger and more educated caregivers use e-learning platforms. We did not explore usability aspects of the e-platform that would further contribute to its limited usage. By offering participants a choice between a printed version and an e-learning platform for educational material delivery, we wanted to ensure that the knowledge needed is delivered to everyone involved in the intervention group in the best user-approachable manner. The preference for physical copies of the manual underscores the necessity of acknowledging various learning modalities and addressing the individualized needs and preferences of study participants. Even in terms of sustainability, research has shown that different dementia post-diagnostic care models can be upgraded and improved by setting an e-learning platform with educational materials for both professionals and carers and tightly connecting them within the social services in the community (Dreier-Wolfgramm et al., 2017). Although we did not test the knowledge of caregivers at T2, the educational materials are currently among the rare informational resources available for family caregivers. From previous studies, we know that family caregivers in Macedonia are not satisfied with the quantity or quality of information they receive about dementia and caregiving upon diagnosis (Taneska et al., 2022). Studies have shown that better dementia knowledge improves confidence in dementia care, which subsequently leads to positive attitudes and a domino effect in the decrease of neuropsychological symptoms in people with dementia and caregiver burden and an improvement of quality of life in caregiver–persons living with dementia dyads (Teichmann et al., 2022). The NOMAD e-learning platform has the potential to enhance knowledge that, over an extended period, could positively impact awareness and attitudes and foster a better understanding of dementia, ultimately leading to improved recognition, treatment, and care, as reported in previous reviews of similar platforms (Klímová et al., 2019; Muirhead et al., 2021).

The problems experienced with recruitment highlight the gaps in diagnosing dementia in North Macedonia, as practitioners often deviate from diagnostic protocols and guidelines, revealing a lack of professional awareness and sufficient knowledge about dementia. As North Macedonia has a poor tradition of conducting clinical and care-related dementia research (Alzheimer Europe, 2023), we have underestimated the impact of inaccurate diagnosis on recruitment. Globally, the milder the dementia, the lower the diagnostic accuracy, which affects practice and research (Van Den Dungen et al., 2011). Additionally, the lower involvement of GPs in the creation and review of care plans due to a lack of time and confidence in supporting people affected by dementia is a further challenge resulting from the insufficient knowledge of dementia. This is compounded by the trend of decline in the number of GPs per 100,000 citizens identified in North Macedonia (Association for Health Education Research HERA, 2018). A lack of knowledge about dementia and clarity of care pathways and time pressures, as well as pressure from families for quick resolutions of behavioral and psychological symptoms of dementia (BPSDs) and discomfort in stepping outside the traditional prescribing role, have been described as barriers to GPs' bigger involvement in non-pharmacological interventions. Contrary to our expectations, GPs were less involved in creating and adjusting the care plan than anticipated. Initially envisioned as a facilitator, the dementia expert emerged as a crucial figure in our care model, as GPs in North Macedonia do not prescribe or de-prescribe dementia drugs or antipsychotics for BPSDs (when pharmacological treatment is needed) without prior patient referral to a secondary or tertiary level. Following recommendations for best-practice primary-led dementia care (Frost et al., 2020a,b), we envisioned collaboration between the dementia professional and the primary care provider. This collaboration allows combining the expertise of the dementia professional with the GP's familiarity with the patient and their overall health. We expected that participation in this service model would also build capacity in GPs and allow continuity of care and sustainability of care plans, which is related to safer prescribing and better outcomes for patients (Delgado et al., 2022). This indicates the necessity of education in dementia care and building capacity among GPs to secure the sustainability and future implementation of this model. The need for training in dementia care has been identified by GPs themselves as they find dementia to be a complex and challenging area as highlighted in Foley et al. (2017) paper on GPs' dementia care educational needs. Increasing knowledge in diagnosing and disclosing dementia, more efficient management of psychological symptoms, signposting to local services, and more support have consistently been identified as GP's educational needs.

The perfect completion of the home visits with all families showed that the intervention was acceptable, but the case notes showed that its acceptability was underpinned by relationship building. We found that the participants were initially apprehensive about the home visits and their purpose besides the explanation provided by the GPs, the research team, and the written information sheet. In addition to the previously mentioned lack of experience with research (Alzheimer Europe, 2023), this apprehension could also be attributed to the novelty of this approach and the unfamiliarity of participants with it. Other studies have found concerns from participants about receiving intervention at home due to fear of judgment from their surroundings or because they did not want to discuss aspects of the care in front of the people with dementia (Webster et al., 2023). In the same study, the facilitators expressed feelings of anxiety and discomfort before entering the participants' homes for the first time. The presence of a social worker in MTs might have caused further discomfort for the participants, potentially because of perceptions of social workers as “judges” of the adequacy of the care they provide. This has been found in other studies of people with dementia in which social workers were deemed as a “threat” to the person's identity, wellbeing, or safety (Wilberforce et al., 2022) and that they had to put significant effort into building relationships and trust with the family. This was reflected in the MTs' case notes, which evidenced that the families were more welcoming after the first visit and after participants recognized the MTs' good intentions. Building trusting relationships has been found to be crucial in improving access to dementia services for underrepresented groups (Brijnath et al., 2021). Although our group was not underrepresented in the conventional sense, the novelty of the service implied that it had not been accessed before. Resistance to novel collaborative care models by families and professionals has been identified as a challenge in their implementation (Galvin et al., 2014).

### 4.2 Outcomes for persons living with dementia

People with dementia experience difficulties not only in cognitive domains but also with everyday activities, social life and engagement, and financial and legal issues. The post-diagnostic management including individually tailored, person-centered, psychosocial, non-pharmacological interventions could prevent or mitigate neuropsychiatric symptoms that are the main reason for long-term use of antipsychotics and institutionalization (Backhouse et al., 2016). In spite of the progressive deteriorating course of the disease, much of the BPSD spectrum could be prevented or mitigated by non-pharmacological interventions and family caregivers' education (Cheng, 2017).

We found a significant reduction in the neuropsychiatric symptoms of people with dementia in the intervention group even after adjusting for their higher prevalence in the control group at baseline. Neuropsychiatric symptoms of dementia are a major cause of worse outcomes for people with dementia and caregivers (Phan et al., 2019). Collaborative case management for dementia care has been found to reduce neuropsychiatric symptoms by Callahan et al. (2006) and Frost et al. (2020a,b). The neuropsychiatric symptoms in dementia are affected by the caregivers' skills (Huang, 2022) and individual caregiver psychoeducation about approaching and managing neuropsychiatric symptoms in dementia has been found to have long-term efficiency in their reduction (Livingston et al., 2005). Non-pharmacological interventions for the reduction of Neuropsychiatric symptoms (NPS) are preferred to antipsychotics due to the lack of adverse effects (Brodaty and Arasaratnam, 2012) and are recommended as the first line of treatment (Ballard and Corbett, 2010). The lack of non-pharmacological treatments results in antipsychotics and benzodiazepines being overprescribed to people with dementia in North Macedonia as shown by the data obtained from the records of people with dementia in the National Health System (Velínov et al., 2024).

Our MTs used a comprehensive assessment to identify and describe the needs and potential causes of neuropsychiatric symptoms in the person with dementia and used a tailored combination of lifestyle and environment changes, caregiver education, and referral to services to tailor addressing these needs, which has been identified as best practice (Kales et al., 2014). Structured approaches to identifying and addressing neuropsychiatric symptoms of dementia (e.g., the Describe, Investigate, Create, Evaluate (DICE) approach), which are people with dementia- and caregiver-centered have been recommended for their management and have the potential to address the burden they cause in caregivers (Eikelboom et al., 2022). Multidisciplinary care involving a care plan developed by more than one healthcare provider has been found highly efficient in reducing depressive symptoms in people with dementia without major depressive disorder (Watt et al., 2021), as well as aggressive and agitated behavior (Watt et al., 2019).

Given the complex nature of neuropsychiatric symptoms in dementia and their multifaceted implications (Phan et al., 2019), it is understandable that their management requires a complex and personalized approach. The involvement of a nurse, a clinical psychologist, a social worker, and the GP in creating and delivering the personalized care plan while supporting the family caregiver and increasing their knowledge of behavioral symptoms and their management allowed a comprehensive approach. It was primarily non-pharmacological and supported the optimization of the pharmacological treatment and management of pain and other conditions by referrals to secondary care. Approaches that combine non-pharmacological and pharmacological treatments in addressing the needs of people with dementia have been recommended as best practice (Kales et al., 2014).

We have also found a significant reduction in the number of healthcare services used by people with dementia in the intervention group. This is in line with previous research showing that interprofessional models of dementia care reduce referrals to specialist services (Michalowsky et al., 2019; Heintz et al., 2020). According to the Macedonian Ministry of Health, behavioral problems and family caregivers' stress are among the most common reasons for seeking medical help (Ministry of Health of Macedonia., 2015). Thus, it is understandable that by targeting caregivers' distress and the neuropsychiatric symptoms of people with dementia and addressing other physical health problems and medications while educating family caregivers on how to manage certain situations and symptoms, NOMAD reduced the use of other services. While we did not assess the cost-effectiveness of the intervention, there is a potential that it reduces the direct and indirect costs of dementia. On one hand, people with dementia use healthcare services across the whole range frequently (Weber et al., 2011), and this contributes to the direct costs of dementia (Cantarero-Prieto et al., 2020). There is evidence that the significant causes of hospitalization for people with dementia are preventable with the appropriate identification and management of risk causes (Gungabissoon et al., 2022). On the other hand, the indirect costs are much higher and attributed to the lack of self-care, loss of work hours, and worsened health in caregivers. NOMAD addresses the latter by reducing depressive symptoms and educating family caregivers and the former by reducing the services used, but a full cost-effectiveness analysis will be necessary to explore this further.

Besides the insignificant reduction in family caregiver burden in the intervention group, the reduction of neuropsychiatric symptoms of dementia can affect the wellbeing of the family caregiver too. Behavioral problems have consistently been identified as predictors of caregiver burden, even more than the cognitive symptoms of dementia (Van Der Lee et al., 2014; Chiao et al., 2015). Considering the bidirectional relationship between caregiver burden and neuropsychiatric symptoms (Isik et al., 2019), reducing the latter has the potential to reduce the levels of burden in family caregivers and subsequently improve the care they provide.

There was no improvement in the ADLs in the intervention group. Non-pharmacological interventions have been found effective in improving ADLs in people with dementia; however, the literature shows that targeted physical activity interventions or occupational therapy (McLaren et al., 2013) or cognitive rehabilitation using functional tasks (Garrido-Pedrosa et al., 2017) have the most effectiveness. While our MTs recommended activities to increase the activity and engagement of people with dementia and reduce risks of falls, we do not have insight into whether and to what extent they were followed. These recommendations differ from targeted interventions (e.g., physical exercise or cognitive stimulation) in which the delivery of the intervention is monitored. Additionally, there is no occupational therapy available in North Macedonia, which means that the MTs could not refer the person with dementia to this service. A systematic review by Mayo-Wilson et al. (Mayo-Wilson et al., 2014) has found that home visits by professionals for people with dementia that include recommendations on improving ADLs are not effective in improving independent living and the efficacy of the interventions depends on the adherence to the recommendations. Additionally, the focus on pharmacological treatment and the lack of dementia friendliness and inclusion Alzheimer Europe (AE) may enhance the prescribed social disengagement in people with dementia in North Macedonia so that the family takes over most of their responsibilities (Low et al., 2017). Furthermore, there is evidence that family caregivers tend to underestimate the functional abilities of people with dementia on proxy measures (Piersol, 2013).

### 4.3 Outcomes for family caregivers

We found that family caregivers in the intervention group had reduced levels of depressive symptoms. The progressive nature of dementia, coupled with the demanding nature of caregiving responsibilities, leads to high levels of stress, depression, and social isolation among family members who serve as informal and unpaid caregivers. Moreover, family caregivers frequently report challenges in balancing caregiving duties with other responsibilities, such as work and personal life, leading to a decline in their own wellbeing. It has been found that providing care over a prolonged time has a significant impact on the caregiver's physical and mental health status (Manzini and Vale, 2020).

As in many developing countries, in North Macedonia, family members of people with dementia are left with no education, help, or support of any kind to live with the challenges of the disease 24/7 for years (Maestre et al., 2023). Not surprisingly, yet very upsetting, 87% of all the family caregivers for people with dementia involved in the study have depressive symptoms with a mean PHQ-9 score of 12, consistent with moderate depression, and 15% fulfill the scoring criteria on PHQ-9 for severe depression. Different studies provide data on the overall prevalence rates of depression and anxiety in family caregivers, from 34% and 44%, respectively, and up to 60% for either depressive or anxiety disorder (Sallim et al., 2015; Cheng, 2017). Our results reveal that the prevalence of depressive symptoms in caregivers is almost double compared to other studies. The burden of informal caregiving is more serious in developing countries where formal services and benefits for people with dementia and caregivers are lacking (Prince et al., 2015; Wimo et al., 2016; Cheng, 2017). Caregiver depression is a perspective not previously explored in North Macedonia and is shedding light on the substantial unmet needs and burdens faced by family caregivers that urgently need to be brought to the attention of policymakers and health authorities; however, previous studies have identified the availability of training and professional consultations among the highest needs of caregivers (Taneska et al., 2022), which are addressed by NOMAD. Depression in family caregivers, accompanied by the family history of dementia, sleep deprivation, a lack of physical activity, and social isolation due to a lack of self-care, free time, and stigma identifies dementia family caregivers as a group with potentially higher risk for developing dementia in later life (Huang, 2022), as midlife depression is recognized as one of the modifiable risk factors for dementia (Livingston et al., 2020).

The main success of the NOMAD study lies in demonstrating the effectiveness of the innovative dementia care model in significantly reducing family caregivers' depressive symptoms in the intervention group, which is in line with previous research (Callahan et al., 2006; Frost et al., 2020a,b). The support provided by the mobile MTs over four home visits, telephone consultations, and their availability to listen to their needs and offer support, paired with provided education and knowledge, shaped the overall outcome. Non-pharmacological, psychosocial interventions, counseling, and education have been shown as beneficial in reducing depressive and anxiety symptoms in family caregivers (Livingston et al., 2013, 2014, 2019; Dickinson et al., 2016).

Studies have shown that BPSDs mutually affect the family caregiver's skills and vice versa are also influenced by the caregiver. This bidirectional relation can easily turn into a vicious cycle of worsening BPSDs due to caregiver exhaustion, burnout, sleep deprivation, and poor mental health, leading to less desirable outcomes for both the people with dementia and the caregiver (Huang, 2022) and lack of social support has been identified as the main contributor of worsened quality of life in Macedonian dementia caregivers' (Taneska, 2020). The results from our study support this interplay, and on one hand, the significant reduction of depressive symptoms in caregivers might be a reason for the significant reduction of the neuropsychiatric symptoms in people with dementia in the intervention group. On the other hand, previous studies have found that neuropsychiatric symptoms play a mediating role in family caregivers' depressive symptoms, so by reducing the neuropsychiatric symptoms in people with dementia, NOMAD may be improving outcomes for family caregivers (Ondee, 2013; Givens et al., 2014). Depressive symptoms may also be targeted by the perceived available support and an increased sense of mastery and confidence in caregiving provided by the psychoeducation and the targeted management of the needs of people with dementia, which has been found in other studies (Galvin et al., 2014).

The burden of the family caregivers is a multiple-layer issue including social isolation, financial strain, and disruption of personal and professional lives, all in all, a demanding experience that poses a threat to the psychological wellbeing and physical health of caregivers (Cheng, 2017). Even though our results did not show a significant reduction in caregiver burden, there is a significant reduction in the caregivers' use of outpatient and inpatient services, which, together with the significant reduction of depressive symptoms in the intervention group, supports the conclusion that NOMAD as a dementia care model that has a positive influence on the overall wellbeing of the family caregivers. We did not find evidence of significant improvement in the quality of life of caregivers in the intervention group and their levels of burden. Quality of life is a multifaceted and complex concept associated with multiple factors coming from the person with dementia and the caregiver (Farina et al., 2017). Although NOMAD addressed some of the aspects of quality of life, such as the depressive symptoms of the caregivers and the neuropsychiatric symptoms of the person with dementia, there are other unmet needs that might contribute to their quality of life. Previously, we have identified that the availability of professional care and respite, as well as day centers, are among the higher needs of family caregivers in Macedonia (Taneska et al., 2022). While NOMAD supports family caregivers in managing care better, they still do not have additional support or opportunities for respite and engagement of the people with dementia in activities, which may contribute to their feelings of burden (Pinquart and Sörensen, 2003a,b), and some authors have found that caregivers' burden is the strongest predictor of quality of life of caregivers (Abdollahpour et al., 2014).

Furthermore, NOMAD did not directly address the lack of dementia-friendly policies, recognition of caregivers' rights, and overall dementia friendliness in Macedonia, as well as the negative attitudes toward dementia, which need longer time and more effort to be improved. Finally, the short version of the ZBI, chosen to reduce the burden of data collection might have not been sensitive enough to capture the change in the levels of burden (Moniz-Cook et al., 2008).

### 4.4 Implementation of NOMAD in the macedonian healthcare system and considerations for practice

NOMAD has the potential to address the significant gap in post-diagnostic care in North Macedonia by combining the most important aspects of post-diagnostic care identified in the literature (Bamford et al., 2021) while also improving access to isolated, less mobile, and rural living people with dementia by offering home visits. This is important as in North Macedonia, older people are more likely to live in rural areas (State Statistic Office, 2022), and the number of older people living alone is increasing due to the increased migration of young people.

Inequalities among countries are seen in each facet of the dementia management process, from awareness and early diagnosis engaging biomarkers through treatment application facilities and infrastructure to creating collaborative post-diagnostic care models and attitudes toward dementia (Maestre et al., 2023). NOMAD, although designed to assess the effectiveness of the dementia care model, revealed gaps, attitudes, and considerations for implementation and offered a more comprehensive perspective on the understanding of dementia in North Macedonia.

The European Dementia Monitor, published at the end of 2023, confirmed the expected inequalities in access to dementia care and treatment across Europe and puts North Macedonia on the lowest scales with no points regarding recognition of dementia as a national policy and research priority, having dementia-inclusive initiatives and communities, and protecting the legal rights of people with dementia and availability of care services (Alzheimer Europe, 2023). The services are fragmented, and there is a lack of support system for families affected by dementia. These are challenging times for the healthcare system and social services regarding dementia management and there are gross differences among the care structure, types, and costs of dementia care for people with dementia across Europe and worldwide, but unlike many countries in South and Southeastern European, most of the countries from Northern Europe have formal care services (Alexopoulos et al., 2020). These disparities are expected to further deepen with the novel disease-modifying treatments and the inequalities among different countries regarding health outcomes, care, and quality of life of people with dementia will become more obvious, creating an even bigger gap than the one already existing (Jönsson et al., 2022, 2023).

While collaborative care interventions with mobile teams may address the lack of post-diagnostic support, the fragmentation of services, and accessibility, their implementation in practice requires strategic planning, and collaboration between healthcare providers, community organizations, and government agencies, as well as adequate funding and training for healthcare professionals. Additionally, raising awareness about dementia and reducing stigma are essential components of supporting families affected by dementia. Furthermore, attitudes and levels of stigma may differ among different groups in society (Alzheimer's Disease International World Alzheimer's Report., 2019). Given that more than 40% of the residents in Macedonia are part of ethnic minority groups, with marked cultural and linguistic differences from the majority population (State Statistic Office, 2022), further adaptation of the model and addressing specific attitudes to dementia may be necessary.

Successful implementation of collaborative care models requires organizational support, care coordination, shared decision-making and support mechanisms, systems for self- and family-led management, as well as adequate electronic and information systems in place (Galvin et al., 2014). While there are long-term system benefits, it requires a high initial investment in increasing and training the workforce and setting up systems, as well as addressing public attitudes. Compared to single professional psychiatry- or neurology-led models, collaborative care models require higher time availability, caring and respect for patients, communication with patients, and inclusion in decision-making (Galvin et al., 2014). As with numerous nations globally, North Macedonia faces challenges in providing comprehensive support for individuals with dementia and their families due to limited resources and competing healthcare priorities. Additionally, the varying preferences of the format of the caregiver manuals indicate that flexibility will need to be offered in how interventions are delivered.

In NOMAD as a dementia post-diagnostic care model, GPs were given a pivotal role, as the central coordinators of the activities and closest to the patient with dementia–caregiver dyad. Yet, the challenges that emerged in terms of recruiting people with dementia and selecting suitable candidates, as well as their limited participation in developing personalized treatment plans, particularly evident from our experience, underscores crucial considerations for the future model of post-diagnostic dementia care in North Macedonia. Although a GP-centric approach is envisioned, it is imperative to complement it with the expertise of specialist neurologists or psychiatrists. This necessity arises due to the lack of sufficient knowledge of GPs, their restricted availability owing to patient overload, and limitations due to local regulations preventing them from prescribing dementia-specific medications or discontinuing therapy prescribed by specialists (Ministry of Health of Macedonia, 2015). The insights gained from the NOMAD experience come as a confirmation of our prior observations from fieldwork and the underrepresentation of dementia-related content in the curriculum for medical students in Macedonia (Faculty of Medicine, 2014). This gap has been identified in HICs and resulted in the development of educational programs to improve awareness of and knowledge about dementia among medical students (Banerjee et al., 2021). With the predicted increase in the number of people with dementia, it will be necessary to include and elevate dementia as a high priority in the educational curriculum for undergraduate medical students and in the curriculum of family medicine specialization.

Besides its potential to decrease direct and indirect costs, without a full cost-effectiveness analysis, it will be difficult to determine how cost-efficient NOMAD is. While we witnessed a reduction in the usage of healthcare services in the intervention group, the opportunity to call the MTs in between visits might have signified that one service was swapped with the other. Given that the MT members were of lower bands than GPs and neurologists, further investigation into the necessary number of MTs to maximize the cost-effectiveness of the model is necessary.

NOMAD has the potential to be improved and implemented as an effective multi-professional dementia post-diagnostic care model that would support family caregivers with education and coordination of services and should be at the forefront of successful dementia management with both short- and long-term impacts.

The innovative care model can inform national dementia strategies and models of care in North Macedonia (included in the National Strategy for Dementia Treatment) and in Germany.

### 4.5 Strengths and limitations

#### 4.5.1 Strengths

NOMAD is the first study to investigate a non-pharmacological, collaborative case management, and psychoeducation model of post-diagnostic care in Macedonia. Its Randomised Control Trial (RCT) design allowed a more robust comparison between the intervention and control group, and the adjusted analysis allowed accounting for other variables that might have affected the results. The variety of participants' demographics in terms of age, gender, living situation, dementia diagnosis, and nationality allowed a better insight into the generalizability of the findings across different groups of participants. The perfect completion of the home visits shows that the intervention is acceptable. The model has the potential to change post-diagnostic management of dementia in North Macedonia.

Although attitudes and stigma surrounding dementia are not easily altered, several studies have shown that positive changes can be expected by increasing knowledge through education (Chang and Hsu, 2020). After completing the NOMAD study intervention, the e-learning platform became accessible to the public. Health professionals, social workers, and caregivers can freely obtain valuable knowledge in the Macedonian language, which significantly enhances the NOMAD study's importance and is our contribution to fighting stigma and changing attitudes toward people with dementia in the country.

#### 4.5.2 Limitations and recommendations for future research

Despite the robust design of the intervention, the study has some limitations. A separate feasibility study would help us identify and manage significant challenges encountered during recruitment and delivery of the study procedures. Cluster randomization was necessary to avoid contamination among participants, but it might have caused clustering effects, besides the use of a random-effects regression. The more common living together with the person with dementia and lesser involvement of other caregivers might have facilitated the implementation of the intervention. Although we have reviewed the MTs' case notes to check if the procedures of delivery were followed, there was a lack of standardized fidelity checks due to the highly personalized nature of the care plans and actions taken. The telephone collection of baseline and follow-up data might have affected the participants' comprehension and motivation to answer the questions, although the measures showed good reliability.

Due to contextual factors discussed earlier (stigma around dementia, unclear legislation about decision-making, and a lack of experience with research participation), we collected only proxy measures for people living with dementia, which may not accurately reflect their experiences. This could also explain the recruitment rate of < 50% as it was the first-ever RCT in dementia care in North Macedonia in addition to the lack of formal support and non-pharmacological treatment, which may have led to mistrust in this approach. We hope this study to lead the way to building a better and more inclusive infrastructure for dementia research in North Macedonia. The limited research capacity meant that we could only interview memory team members about their experiences with delivering the model (which will be reported elsewhere) but not the participants, so we lacked in-depth insight into their subjective experiences and perceptions about the model.

A process evaluation in the future might be necessary to determine the main drivers of the outcomes before implementing it into practice. As this study was exploring the feasibility of the model, further research should include focused implementation studies. Due to the smaller number of participants from ethnic and minority groups, we did not stratify the results by ethnicity. However, further cultural and linguistic adaptations to the intervention may be necessary to better cater to the needs of minority groups given it was delivered in Macedonian.

## 5 Conclusion

As an innovative multicomponent and interprofessional model of dementia care, NOMAD has been found acceptable by participants and effective in reducing caregivers' depressive symptoms and neuropsychiatric symptoms and service usage in people with dementia. Improving these outcomes for people affected by dementia can have long-lasting effects on an individual and a system level. Due to its flexibility and comprehensiveness, it has the potential to bridge the existing gaps in provisions for post-diagnostic dementia care and support for families in North Macedonia, reduce the burden on healthcare services, and inform policy. The challenges encountered on-site will inform necessary adaptations in the model for its implementation in practice including ensuring adherence to diagnostic protocols, building GPs' capacity, and building relationships with service users. The takeaways from these projects can be used by countries facing similar challenges in developing their policies and management of dementia.

## Data availability statement

The raw data supporting the conclusions of this article will be made available by the authors, without undue reservation.

## Ethics statement

Prior to recruitment and data collection, the study was approved by the Ethical Committee of the Medical Faculty at the University Ss Cyril and Methodius in Skopje, North Macedonia (Ref. number:03-1260/5). Application to access the Macedonian e-health data regarding patients diagnosed with dementia were submitted to the Department for E-Health at the Ministry of Health (www.mojtermin.mk) and was granted in January 2024. A written informed consent procedure with the legal proxy of the people with dementia was the basis for study participation. The study was conducted in compliance with European data protection guidelines.

## Author contributions

GN: Conceptualization, Investigation, Supervision, Writing – original draft, Writing – review & editing. MT: Data curation, Methodology, Writing – original draft, Writing – review & editing. AN: Conceptualization, Investigation, Project administration, Supervision, Writing – review & editing. JF: Data curation, Funding acquisition, Project administration, Resources, Supervision, Writing – review & editing. SI: Investigation, Supervision, Writing – review & editing, Methodology, Project administration. AI: Data curation, Formal analysis, Software, Visualization, Writing – review & editing. VD: Data curation, Formal analysis, Software, Visualization, Writing – review & editing. LN: Data curation, Investigation, Writing – review & editing. MM: Data curation, Investigation, Writing – review & editing. BJ: Data curation, Software, Writing – review & editing. IC: Data curation, Software, Writing – review & editing. SH: Methodology, Project administration, Writing – review & editing. VD: Project administration, Supervision, Writing – review & editing. TG: Supervision, Writing – review & editing. AK: Funding acquisition, Methodology, Project administration, Resources, Supervision, Visualization, Writing – original draft.

## Footnotes

^1^ https://demencija.mk

^2^ https://indeed-project.eu/

^3^ https://www.interreg-danube.eu/approved-projects/indeed

## In memoriam

This article is dedicated to the memory of Prof. Alexander Kurz, who died on 29 January 2023.

## Supplementary material

Supplementary Figure 1 Medication prescriptions in people with Alzheimer's disease in 2018, 2019, and 2022.
