# Supplementary figures and images for: North Macedonia interprofessional dementia care (NOMAD) – personalized care plans for people with dementia and caregiver psychoeducation delivered at home by interprofessional teams
Source: Front Dement. 2024 Apr 10;3:1391471. doi: 10.3389/frdem.2024.1391471 (PMC11285573; doi:10.3389/frdem.2024.1391471)

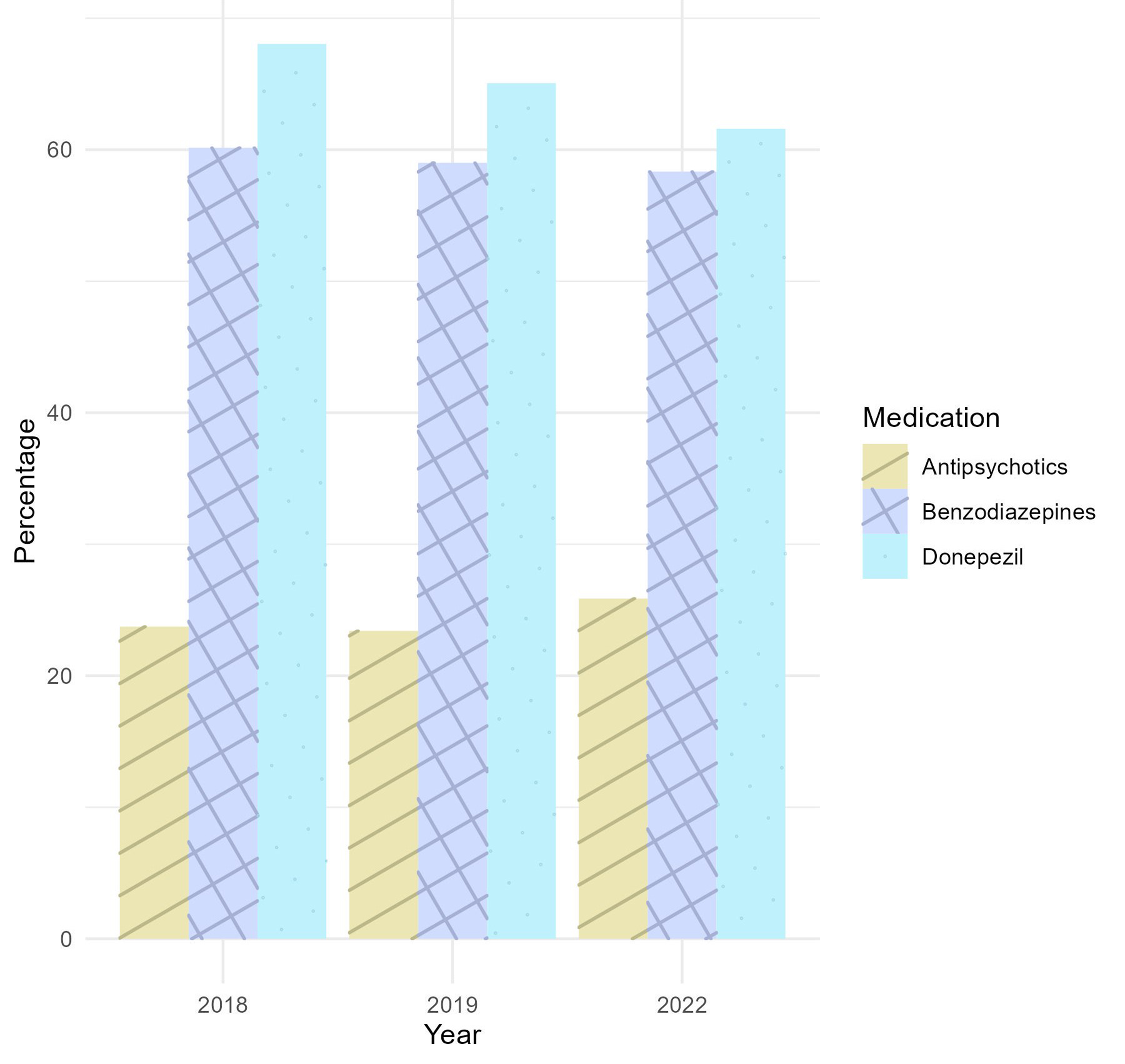

Supplement: Supplementary Figure 1 — Medication prescriptions in people with Alzheimer's disease in 2018, 2019, and 2022. [file Image_1.jpeg]
